# Chronic Hydrocephalus Cured by a Fracture of the Skull

**Published:** 1838

**Authors:** 


### Art. III.—Chronic Hydrocephalus, cured by a fracture of the skull.

A child 5 years old, with every symptom of commencing hydrocephalus, and likely to be soon severely attacked, was violently kicked and thrown down by a cow.

Dr. Hofling being called, observed a gaping fracture of the frontal bone. The child had vomited, but that symptom had ceased, and there was no decided sign of general or cerebral re-action. Antiphlogistic treatment and a simple dressing fulfilled every indication. Changing the dressing, all the lint was found soaked with a clear limpid inodorous fluid—which still flowed from the wound—and continued very abundant for eight days—and then gradually diminished. When it ceased, the cicatrization was rapid, and the child was soon well. It is now three years since this happy accident, the general health is very good, and the head is nearly reduced to its normal size.

Dr. Hofling closes his account by blaming the general inactivity of surgeons, in the treatment of Hydrocephalus. The possibility of curing this dreadful disease by paracentesis has been amply demonstrated. Besides we must remember the adage,—*In extremis morbis melius anceps remedium quam nullum*, which has induced Dr. H. to prescribe the evacuation of the cerebral fluid, by degrees, with successive punctures of a needle or a very small trochar.—*Ibid.*

